# Effectiveness of the antiviral medications azvudine and nirmatrelvir-ritonavir in treating COVID-19 in patients with hematological malignancies

**DOI:** 10.1016/j.clinsp.2024.100406

**Published:** 2024-07-25

**Authors:** Zheng Zeng, Fangyuan Li, Mingli Zhong, Ling Zhu, Wei Chen, Xiaotao Wang

**Affiliations:** a Department of Pharmacy, Affiliated Hospital of Guilin Medical University, Guilin, China; b School of Pharmacy, Guilin Medical University, Guilin, China; c Department of Hematology, Affiliated Hospital of Guilin Medical University, Guilin, China

**Keywords:** Hematologic malignancies, Nirmatrelvir-ritonavir, Azvudine, COVID-19

## Abstract

**Background:**

Patients with Hematological Malignancies (HM) are at a high risk of mortality from Coronavirus disease 2019 (COVID-19). The available antivirals were different between China and other countries. In China, azvudine was obtained for emergency use to treat adult COVID-19 patients with moderate symptoms in July 2022. While nirmatrelvir-ritonavir was well-known and used in many countries. The purpose of the present study was to assess whether there was any difference in the efficacy and safety of the two drugs.

**Methods:**

This study was a prospective observational study of patients with HM who developed COVID-19. Patients were divided into three treatment groups: nirmatrelvir-ritonavir, azvudine, and observation. Treatment outcomes, first nucleic acid test negative time, hospitalization time, and the conversion rate of mild or moderate disease to severe disease were recorded.

**Results:**

First nucleic acid test negative time (23.5 days vs. 34 days, p = 0.015), hospitalization time (p = 0.015), and conversion rate (31.8 % vs. 8 %, p = 0.046) were statistically different between the nirmatrelvir-ritonavir and observation groups. First nucleic acid test negative time (20 days vs. 34 days, p = 0.009) and hospitalization time (p = 0.026) were statistically different between the azvudine and observation groups. ECOG score and liver disease were significantly associated with the conversion rate from mild or moderate disease to severe disease using multivariate analysis (p < 0.05).

**Conclusions:**

The authors found no significant differences existed in outcome measures between patients with HM and COVID-19 who were treated with nirmatrelvir-ritonavir or azvudine.

## Highlights

• In the present study, the authors found a new antiviral drug-azvudinea used in patients with hematological malignancies, is as effective and safe as the well-known nematve-litonavir.
• There have been few clinical trials using azvudine to date, the present study may serve as a suggestion that the treatment was effective.
• In the present study the authors found that anti-SARS-CoV-2 vaccines show significantly less robust efficacy in eliciting an immune response in HM patients than observed in the general population, suggesting that vaccination may be of little value in HM patients.
• In the present study, the authors found a new antiviral drug-azvudinea used in patients with Hematological Malignancies (HM), is as effective and safe as the well-known nematve-litonavir.

## Introduction

The first case of a novel acute respiratory syndrome coronavirus, named SARS-CoV-2 by the WHO, was first reported in China and spread rapidly around the globe in late December 2019. As of December 8, 2022, China ended the requirement for mass COVID-19 testing and later downgraded its management of the disease from Class A to Class B on January 8, 2022. Following the optimization of the antivirus strategy, the COVID-19 epidemic peaked in late December 2022. Patients with HM are at a high risk of developing severe COVID-19, and coincident COVID-19 in patients with HM is associated with high morbidity and mortality due to the systemic immunosuppressive state that is caused by both HM and anticancer treatments.^1^^,^^2^ Thus, severe COVID-19 infections in HM patients may affect clinical outcomes and life expectancy. To date, fewer reports on antiviral therapies in patients with COVID-19 and HM have been published than reports in patients with COVID-19 without HM.

Antivirals available for COVID-19 treatment vary between China and other countries and include nirmatrelvir-ritonavir and azvudine. In China, nirmatrelvir-ritonavir has been granted conditional approval since February 2022 for use in adults with mild to moderate COVID-19 symptoms who are at high risk for progression to severe disease. However, access to nirmatrelvir is limited worldwide and its effectiveness depends on ritonavir,^3^ which has multiple drug-drug interactions, warranting specialized assessment before its prescription.^3^^,^^4^ Azvudine was initially developed as a broad-spectrum antiviral agent, and its emergency use to treat adult patients with moderate COVID-19 symptoms was obtained in July 2022.^5^ Extensive data on the use of nirmatrelvir-ritonavir for the treatment of COVID-19 exists, but there is limited clinical information on the use of azvudine for the treatment of COVID-19. To provide a useful reference for the treatment of COVID-19 in patients with HM, the treatment effects of nirmatrelvir-ritonavir and azvudine in patients with COVID-19 and HM were compared in the present study.

## Materials and methods

### Study design

This study was a single-center, open-label, prospective observational study designed to evaluate the efficacy and safety of adding nirmatrelvir-ritonavir or azvudine to the current, standard conventional therapy used for the treatment of patients with HM and COVID-19. COVID-19 cases were confirmed using the diagnostic criteria noted within the “Scheme for Diagnosis and Treatment of 2019 Novel Coronavirus Pneumonia (The 10^th^ Trial Edition)”. Patients meeting the inclusion and exclusion criteria were divided into three groups: nirmatrelvir-ritonavir, azvudine, and observation. This clinical trial was not blinded. The time to market of nirmatrelvir-ritonavir was February 2022. Azvudine was first approved in July 2021 to treat HIV patients and was also found to be promising in treating COVID-19. Data were collected from December 2022 to January 2023 in the Department of Hematology, Guilin Medical College Hospital.

### Patients

Adult patients with HM and laboratory-confirmed COVID-19 at the Guilin Medical College Hospital were eligible for inclusion. Patients with a diagnosis of leukemia, myeloma, lymphoma, myelodysplastic syndrome, primary thrombocythemia, and primary myelofibrosis, positive nucleic acid test, 18 years of age or older, and study subjects who met the diagnostic criteria for new coronary pneumonia were included in this study.

Pregnant women, patients with a history of drug allergy or hypersensitivity, and patients with other conditions that investigators judged necessary for exclusion were excluded from the study.^6^^,^^7^

### Treatment protocols

Patients in the nirmatrelvir-ritonavir group were given 300 mg nematovir (2 tablets) and 100 mg (1 tablet) ritonavir twice daily for five days in addition to conventional therapy for COVID-19. Patients in the azvudine group were given 5 mg azvudine (1 tablet) once daily for seven days in addition to conventional therapy for COVID-19. Patients in the observation group received conventional therapy only. All conventional treatments were in accordance with the Chinese Diagnosis and Treatment Protocol for COVID-19 (Version 10),^8^ which included supportive therapy (oxygenation), hormonal therapy, and symptomatic treatment. The outcomes of patients pre- and post-treatment were recorded.

### Efficacy outcomes

The primary outcome was all-cause progression of mild or moderate disease to severe or critical COVID-19. Secondary outcomes included the first nucleic acid negative testing time and hospitalization time (discharge criteria were developed using the “Chinese Diagnosis and Treatment Protocol for COVID-19, Version 10”).^9^ Demographic characteristics and the baseline status of patients infected with COVID-19 were collected with respect to gender, age, Body Mass Index (BMI), vaccination status, primary disease, Charlson Comorbidity Index (CCI score), ECOG score (physical status rating scale), disease grade (mild, moderate, severe, and critical as described in the “Chinese Diagnosis and Treatment Protocol for COVID-19, Version 10”), medication status (chemotherapy regimen, enzyme inducers, antimicrobials, and hormones), and laboratory indicators of patients pre- and post-treatment were recorded. BMI was calculated by the investigators as weight in kilograms divided by the square of the height in meters. Obesity was defined as a BMI ≥ 30 kg/m^2^. The CCI score was quantified based on the patient's comorbidities using the CCI scale.

### Statistical methods

SPSS v.23.0 was used to statistically analyze the data, with categorical variables predicted by frequencies and percentages and quantitative variables expressed as mean ± Standard Deviation (SD) or median (range). Baseline characteristics were compared using a Pearson × 2 test for categorical variables and an independent-samples *t*-test for continuous variables. Normality was verified using the Shapiro-Wilks test and histograms. A paired *t*-test was used for normally distributed continuous variables and Wilcoxon rank-sum test for non-normally distributed continuous variables. The clinical efficacy of nirmatrelvir-ritonavir and azvudine was assessed by the Cox proportional hazards mode to investigate the effect of covariates at baseline on the primary outcome. Variables with p < 0.1 were considered for multivariate analysis. A multivariable Cox regression model was calculated using the LR forward method, and only those variables that were statistically significant were displayed. The conversion rate of mild or moderate disease to severe disease, first nucleic acid testing time, and hospitalization time were analyzed using Kaplan-Meier survival charts. Differences were considered significant at p < 0.05.

## Results

### Study sample

A total of 64 patients with confirmed HM and COVID-19 were included in this study. Twenty-two patients received conventional therapy + nirmatrelvir-ritonavir, 17 patients received conventional therapy + azvudine, and 25 patients received only conventional therapy. Patient demographic and clinical characteristics are shown in Table 1. Men (n = 33, 51.6 %) had a slightly higher prevalence of HM and COVID-19 than Women (n = 31, 48.4 %). Patients aged ≥ 60 years and < 60 years were equally represented. The largest subgroup of HM was patients with lymphoma (n = 16, 25 %), followed by patients with AML (n = 15, 23.4 %) and multiple myeloma (n = 12, 18 %). Twenty-eight patients (43.8 %) had received chemotherapy within one month prior to a positive COVID-19 nucleic acid test. More than 80 % of patients with COVID-19 and HM in the nirmatrelvir-ritonavir group reached moderate (n = 9, 40.9 %) and severe (n = 9, 40.9 %) diagnoses. More than 50 % of patients in the aziridine group reached the moderate (n = 7, 41.2 %) and severe (n = 4, 23.5 %) diagnoses. More than half of the patients in the observation group were considered mild (n = 13, 52 %). Twenty-five patients (39.1 %) had not received the neo-crown vaccine, and 23 patients (35.9 %) had received three doses of the neo-crown vaccine. Except for neo-crown typing (p = 0.014), no statistical significance existed between the three treatment groups in terms of gender, age, comorbidities, primary HM subgroup, COVID-19 positive nucleic acid test, time to last chemotherapy, and number of neo-crown vaccinations (p > 0.05).

**Table 1:** Patient characteristics.

**Characteristic**	**Total** **(n** = **64)**	**Nirmatrelvir- ritonavir group** **(n** = **22)**	**Azvudine group** **(n** = **17)**	**Observation group** **(n** = **25)**	**p**
Sex, n (%)					
Female	31 (48.4)	11 (50)	6 (35.3)	14 (56)	0.413
Male	33 (51.6)	11 (50)	11 (64.7)	11 (44)	
Age, n (%)					
≥ 60 years old	32 (50)	11 (50)	11 (64.7)	10 (40)	0.291
< 60 years old	32 (50)	11 (50)	6 (35.3)	15 (60)	
Comorbidities, n (%)					
Chronic heart disease	13 (20.3)	5 (22.7)	4 (23.5)	4 (16)	0.078
Chronic lung disease	5 (7.9)	1 (4.5)	3 (17.6)	1 (4)	
Diabetes mellitus	9 (14.2)	7 (31.8)	0	2 (8)	
Liver disease	12 (19)	2 (9.1)	4 (23.5)	6 (24)	
Obesity	1 (1.6)	1 (4.5)	0	0	
Kidney damage	4 (6.3)	2 (9.1)	0	2 (8)	
Smoking history	6 (9.5)	1 (4.5)	1 (5.9)	4 (16)	
No risk factor identified	24 (38.1)	8 (36.4)	5 (29.4)	11 (44)	
Baseline HM, n (%)					
Acute lymphocytic leukemia	3 (4.7)	1 (4.5)	0	2 (8)	0.168
Chronic lymphocytic leukemia	2 (3.1)	0	2 (11.8)	0	
Acute myelogenous leukemia	15 (23.4)	6 (27.3)	4 (23.5)	5 (20)	
Chronic myelogenous leukemia	5 (7.8)	2 (9.1)	1 (5.9)	2 (8)	
Myelodysplastic syndrome	8 (12.5)	1 (4.5)	1 (5.9)	6 (24)	
Lymphoma	16 (25)	7 (31.8)	4 (23.5)	5 (20)	
Primary thrombocythemia	2 (3.1)	0	2 (11.8)	0	
Primary myelofibrosis	1 (1.6)	0	1 (5.9)	0	
Multiple myeloma	12 (18)	5 (22.7)	2 (11.8)	5 (20)	
Last chemotherapy before COVID-19, n (%)					
> 3 months	16 (25)	3 (13.6)	5 (29.4)	8 (32)	0.117
Within 3-months	20 (31.3)	5 (22.7)	8 (47.1)	7 (28)	
Within 1-month	28 (43.8)	14 (63.6)	4 (23.5)	10 (40)	
Disease grade, n (%)					
Mild	23 (35.9)	4 (18.2)	6 (35.3)	13 (52)	0.014
Moderate	27 (42.2)	9 (40.9)	7 (41.2)	11 (44)	
Severe	14 (21.9)	9 (40.9)	4 (23.5)	1 (4)	
Number of neo-crown vaccinations					
0	25 (39.1)	8 (36.4)	7 (41.2)	10 (40)	0.887
1	2 (3.1)	1 (4.5)	1 (5.9)	0	
2	13 (20.3)	5 (22.7)	3 (17.6)	5 (20)	
3	23 (35.9)	7 (31.8)	6 (35.3)	10 (40)	
4	1 (1.6)	1 (4.5)	0	0	

### COVID-19 predictors of the conversion rate of mild or moderate disease to severe disease in patients with HM

To predict factors associated with the conversion rate to severe disease in 64 patients, Cox regression analysis models were used for univariate and multivariate analyses (Table 2). Risk variables included sex, age, BMI, ECOG score, CCI score, SPO_2_, comorbidities, primary HM, typing, chemotherapy time, and the number of COVID-19 vaccinations. In univariate analysis, age (HR = 0.204, 95 % CI 0.047−0.882, p = 0.033), ECOG score (HR = 1.82, 95 % CI 0.911−3.625, p = 0.09), and liver disease (HR = 16.189, 95 % CI 1.638−160.042, p = 0.017) were significantly associated with the conversion rate to severe disease. In multivariate Cox regression analysis, ECOG score (HR = 2.284, 95 % CI 1.036–5.038, p = 0.041) and liver disease (HR = 25.281, 95 % CI 2.225–287.193, p = 0.009) were independent influencing factors.

**Table 2:** Univariate and multivariate Cox regression analysis of the overall conversion rate of mild or moderate disease to severe disease in 64 patients with COVID-19 hematological malignancies.

**Indicators**	**Overall conversion rate to severe disease**			
	**Univariate analysis**		**Multivariate analysis**	
	**HR (95** % **CI)**	**p**	**HR (95** % **CI)**	**p**
Sex (Male/Female)	0.983 (0.29–3.33)	0.979		
Age	0.204 (0.047–0.882)	0.033		
BMI	0.972 (0.752–1.256)	0.827		
CCI score	1.045 (0.818–1.335)	0.726		
ECOG score	1.820 (0.911–3.625)	0.09	2.284 (1.036−5.038)	0.041
Finger oxygen saturation	0.951 (0.873–1.036)	0.254		
Chronic heart disease	0.644 (0.199–2.086)	0.463		
Chronic lung disease	0 (0)	0.986		
Diabetes mellitus	0.828 (0.24–2.864)	0.766		
Liver disease	16.189 (1.638–160.042)	0.017	25.281 (2.225–287.193)	0.009
Obesity	0 (0)	0.991		
Kidney damage	0.713 (0.088–5.747)	0.751		
Smoking history	1.651 (0.34–8.007)	0.534		
No risk factor identified	0.307 (0.038–2.462)	0.266		
Baseline HM	1.022 (0.826–1.264)	0.842		
Last chemotherapy before COVID-19	1.502 (0.654–3.447)	0.337		
Disease grade	1.123 (0.299–4.210)	0.864		
Number of neo-crown vaccinations	1.331 (0.845–2.096)	0.217		
Whether to receive the neo-crown vaccine	1.714 (0.482–6.099)	0.405		

BMI, Body Mass Index; CCI score, Charlson Comorbidity Index; ECOG score, Physical Status Rating Scale; HR, Hazard Ratio; CI, Confidence Interval.

### Analysis of general data of mortality and conversion rate of mild or moderate disease to severe disease

Mortality and conversion rate of mild or moderate disease to severe disease were higher in patients over 60 years old compared to those under 60 years old (Table 3), suggesting that age may be a variable influencing death or conversion to severe disease in patients with COVID-19 and HM (p = 0.033) (Table 2). Moderate disease patients (n = 6, 23.1 %) and severe disease patients (n = 5, 33.3 %) with COVID-19 were more likely to have disease progression. There were three deaths (11.5 %) and six referrals (23.1 %) among patients who had not received the neo-crown vaccine and two deaths (5.3 %) and seven referrals (18.4 %) among patients who had received the neo-crown vaccine. Two patients (9.1 %) in the nirmatrelvir-ritonavir group died, and seven patients (31.8 %) progressed to severe disease. Three patients (17.6 %) in the azvudine group died, and four patients (23.5 %) progressed to severe disease. No patients in the observation group died, but two patients (8 %) progressed to severe disease. The lower mortality and conversion rates in the observation group compared to the medicated groups were considered to be due to the uneven severity of COVID-19 infection in the three groups prior to treatment.

**Table 3:** Statistical analysis of the overall mortality rate and conversion rate of mild or moderate disease to severe disease.

**Baseline HM**	**Died, n (%)**	**Transferred to Critical Care, n (%)**
Acute lymphocytic leukemia	0	0
Chronic lymphocytic leukemia	0	0
Acute myelogenous leukemia	1 (6.7)	3 (20)
Chronic myelogenous leukemia	0	0
Myelodysplastic syndrome	1 (11.1)	2 (22.2)
Lymphoma	1 (6.3)	4 (25)
Primary thrombocythemia	1 (100)	1 (100)
Primary myelofibrosis	0	0
Multiple myeloma	1 (8.3)	3 (25)
Age		
≥ 60 years old	2 (3.1)	5 (7.8)
< 60 years old	3 (4.7)	8 (12.5)
Disease grade		
Mild	1 (4.3)	2 (8.7)
Moderate	2 (7.7)	6 (23.1)
Severe	2 (13.3)	5 (33.3)
Treatment method		
Nirmatrelvir-ritonavir group	2 (9.1)	7 (31.8)
Azvudine group	3 (17.6)	4 (23.5)
Observation group	0	2 (8)
Neo-crown vaccination		
Not vaccinated	3 (11.5)	6 (23.1)
Already vaccinated	2 (5.3)	7 (18.4)

### Comparison of laboratory index results pre- and post-treatment

Comparative results of laboratory test data pre- and post-treatment in patients are presented in Table 4. CRP and IL-6 levels were significantly lower in the medication treatment group after five to seven days of treatment (p < 0.05). CRP was significantly lower in the observation group after conventional treatment (p < 0.05), however, the decreases in CRP and IL-6 were not as great as those observed in the medication treatment group (Table 4).

**Table 4:** p-values of laboratory indicators pre- and post-treatment in the medication and observation groups.

**Parameters**	**Medication group (mean ± SD)**			**Observation group (mean ± SD)**		
	**Pre-treatment**	**Post-treatment**	**p**	**Pre-treatment**	**Post-treatment**	**p**
WBC (× 10^9^/L)	14.5±52.17	14.4±58.1	0.96	8.6±17.1	13.4±22.5	0.201
LYMPH (× 10^9^/L)	4±18.2	4.4±20.2	0.45	1.1±0.6	9.1±22.1	0.798
NE (× 10^9^/L)	4±6	2.7±3.1	0.139	3.7±4.5	6.8±9.5	0.882
PCT (μg/L)	5.6±13.6	2.7±4.3	0.686	14.7±17.2	9.2±8.6	0.23
CRP (mg/L)	86±78.9	16.9±24.3	<0.001	41.3±57.3	18.9±22.9	0.028
IL-6 (pg/mL)	46.9±73.2	29.2±53.3	0.033	28.6±16.6	20.1±22.7	0.102
CK-MB (ng/mL)	15.8±17	21.6±16.2	0.001	11.7±4.4	18.6±10.9	0.005
PT (s)	13±1.5	13.9±2.5	0.035	12.8±2.7	13.2±3.8	0.644
APTT (s)	32.5±6	26.1±4.8	<0.001	28.1±4.6	24.3±5.7	0.045
D-D2 (μg/L)	2.3±2.4	2.1±1.3	0.664	2.7±4.7	2.5±1.4	0.053
T-BIL (μmoL/L)	12.1±16.1	12±17.2	<0.001	8.6±4.3	7.9±4.3	0.465
ALT (U/L)	28±27.3	26.7±20.8	0.723	24.4±21.8	26.5±7.5	0.115
AST (U/L)	36.2±43.3	39.5±54.9	0.657	28.2±17.5	27.2±8.8	0.548
CCR (mL/min)	101.7±120.8	78.5±30.5	0.8	91.1±44.4	75.2±24	0.037

WBC, White Blood Cell; LYMPH, Lymphocytes; NE, Neutrophil; PCT, Procalcitonin; CRP, C-Reactive Protein; IL-6, Interleukin-6; CK-MB, Creatine Kinase Isoenzyme; PT, Plasma Prothrombin Time; APTT, Activated Partial Thromboplastin Time; D-D2, D-Dimer; TBIL, Total Bilirubin; ALT, Alanine Aminotransferase; AST, Aspartate Aminotransferase; CCR, Creatinine Clearance.

### Efficacy assessment

**Figure 1:**
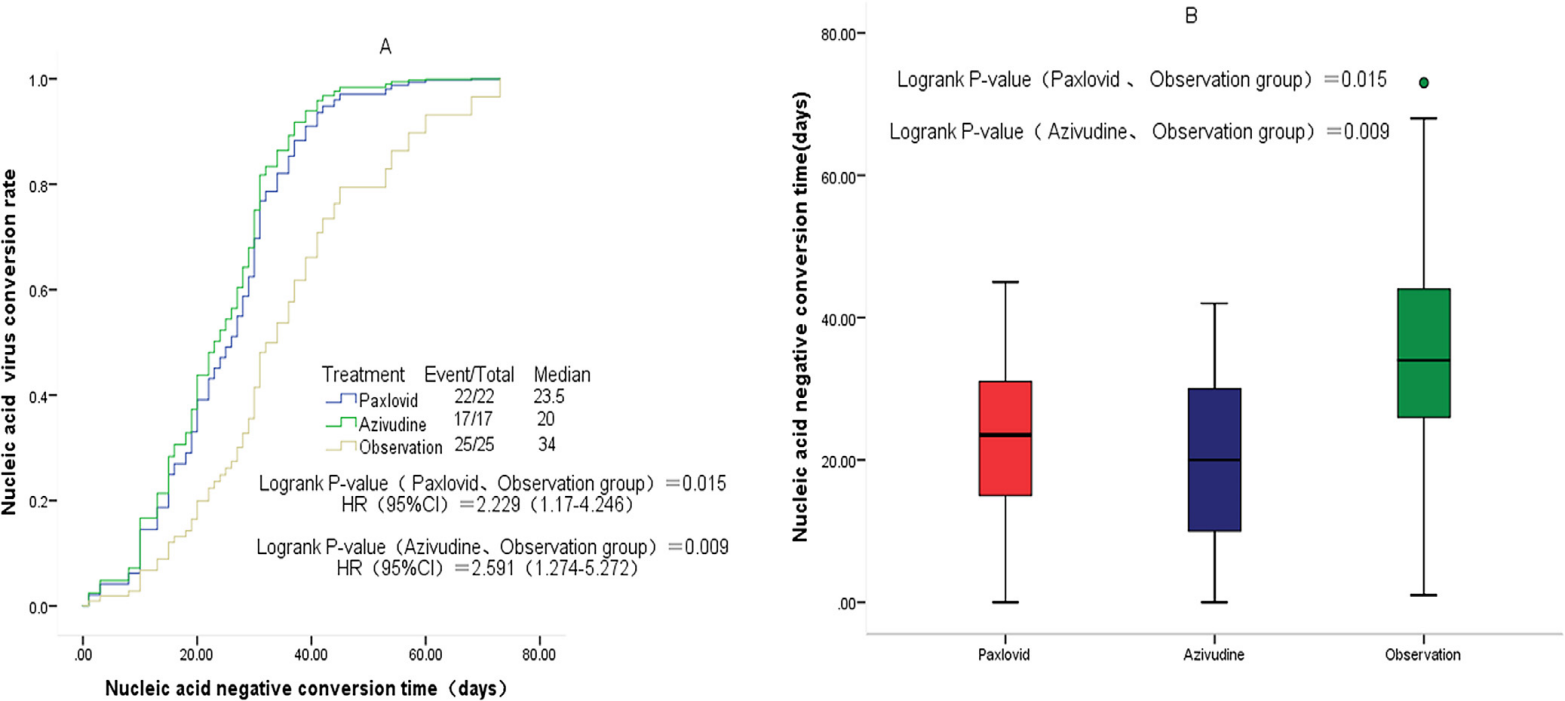
Comparison of COVID-19 nucleic acid negative conversion time process between the three treatment groups. (A) Survival curves of the time process of nucleic acid negative conversion between the three treatment groups. (B) First nucleic acid testing time in the three groups of patients after treatment.

**Figure 2:**
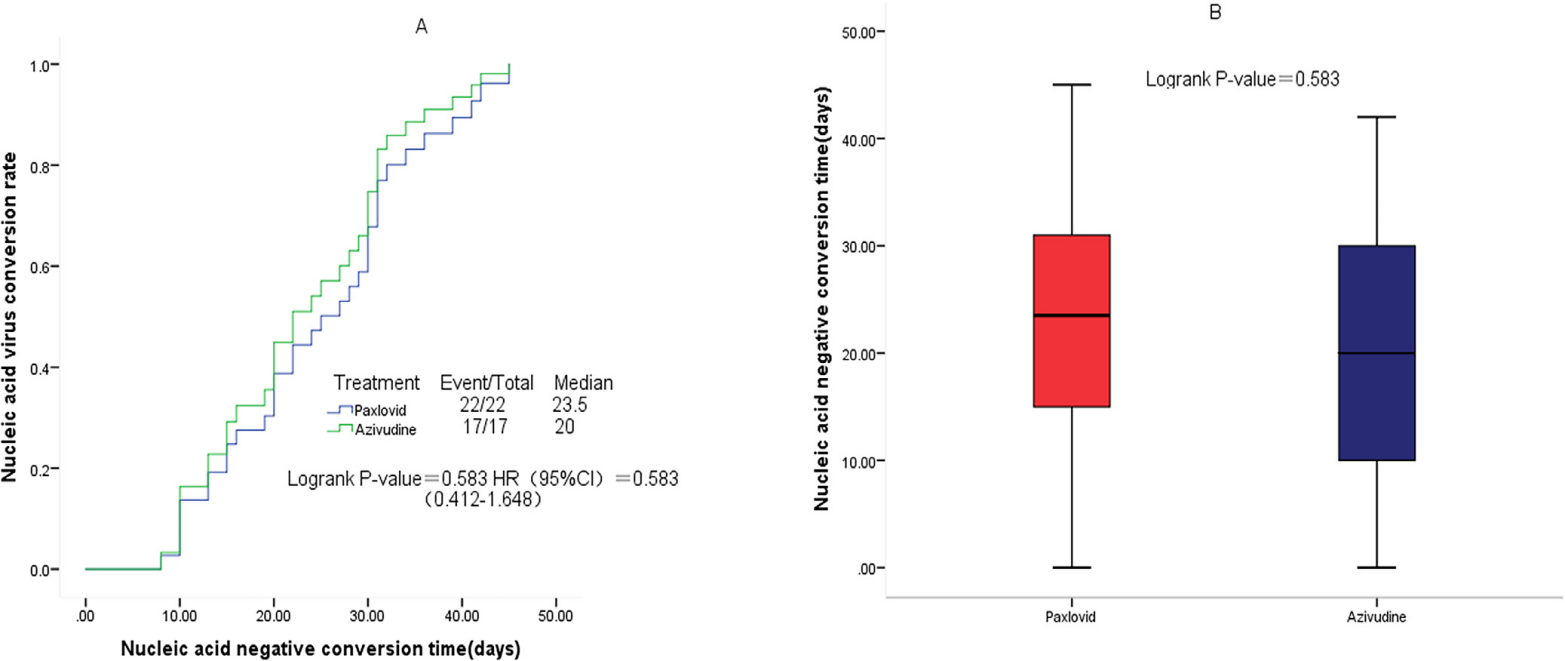
Comparison of COVID-19 nucleic acid negative conversion time process between the nirmatrelvir-ritonavir and azvudine groups. (A) Survival curves of the time process of nucleic acid negative conversion between the two treatment groups. (B) First nucleic acid testing time in both treatment groups.

**Figure 3:**
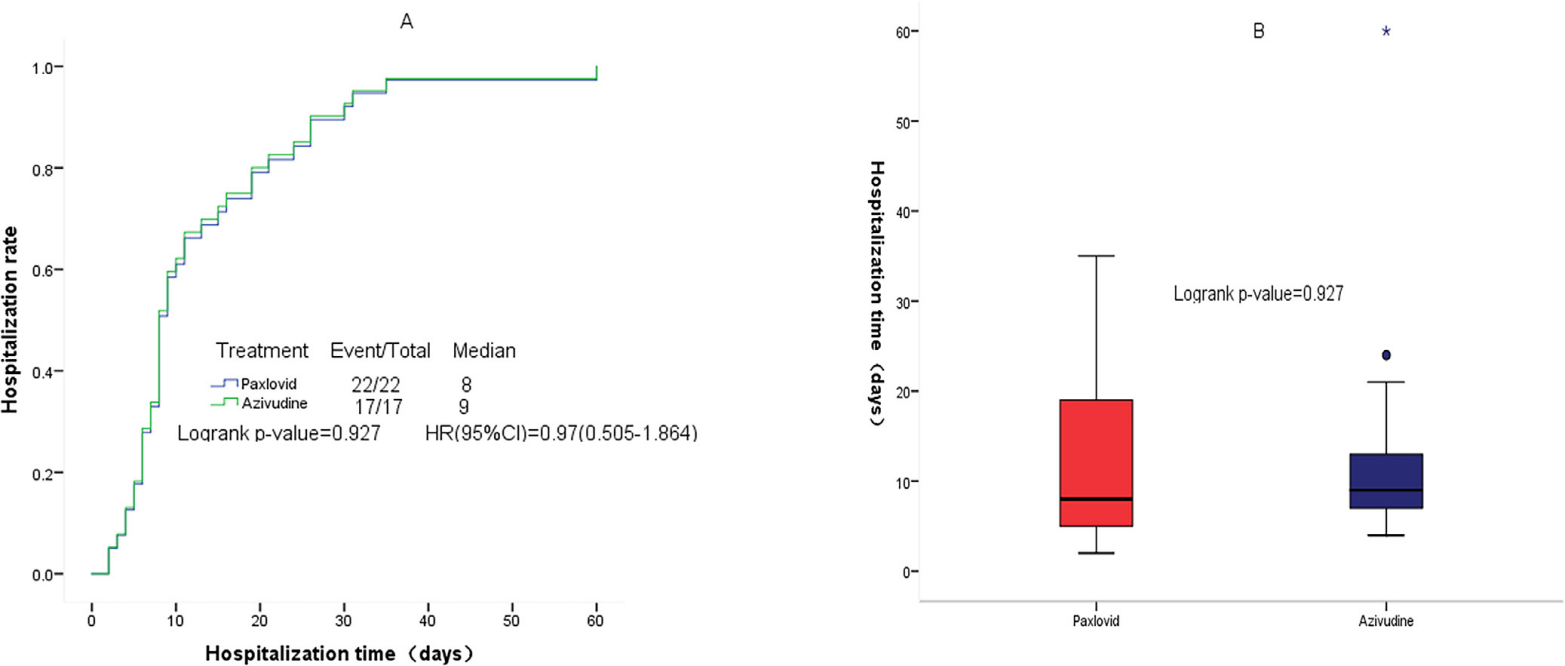
Comparison of hospitalization time process between the nirmatrelvir-ritonavir and azvudine groups. (A) Survival curves of the hospitalization time process between the two treatment groups. (B) Hospitalization time between the two treatment groups.

**Figure 4:**
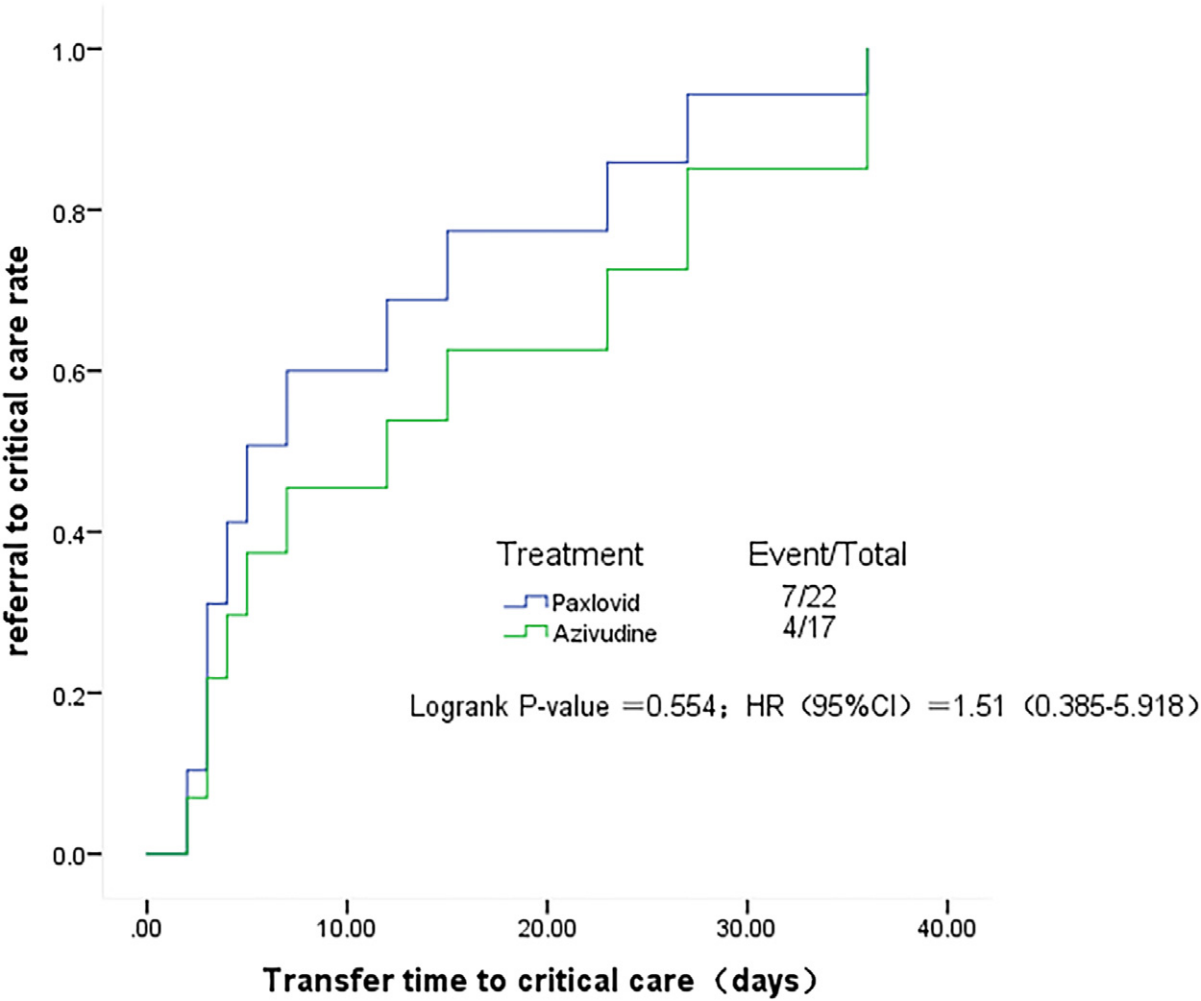
Comparison of the time process of conversion of mild or moderate disease to severe disease in the nirmatrelvir-ritonavir and azvudine groups.

Compared with the observation group, patients in the nirmatrelvir-ritonavir group (23.5 days vs. 34 days, p = 0.015) and azvudine group (20 days vs. 34 days, p = 0.009) had a shorter median time to first negative nucleic acid test (Fig. 1). No significant differences were found in the time to first negative nucleic acid test (23.5 days vs. 20 days, p = 0.583) (Fig. 2), hospitalization time (8 days vs. 9 days, p = 0.927) (Fig. 3), and rate of conversion of mild or moderate disease to severe disease (p = 0.554) between the nirmatrelvir-ritonavir and azvudine groups (Fig. 4).

### Security assessment

There was no significant difference in the incidence of adverse events between the three groups (p > 0.05). Overall, five patients died. No patients in the two groups receiving nirmatrelvir-ritonavir or azvudine experienced allergic reactions or functional impairment of the heart, liver, kidneys, or other organs during the study period.

## Discussion

COVID-19 has been shown to cause damage to the respiratory tract, nervous system, liver, heart, esophagus, kidneys, bladder, and jejunum, with patients diagnosed with severe COVID-19 developing respiratory distress after one week of symptom onset. COVID-19-induced respiratory distress can rapidly progress to acute respiratory distress syndrome, septic shock, and multi-organ failure and lead to other serious complications that endanger the patient's life.^9^ The current prospective observational study explored the therapeutic efficacy of two antiviral agents, nirmatrelvir-ritonavir and azvudine, while predicting variables for the progression of mild or moderate disease to severe or critical neo-crown pneumonia in patients with COVID-19 and concomitant HM.

According to a large survey of patients with COVID-19 and HM conducted across 132 hematology institutions in Europe, the demographic and clinical characteristics of 3,801 active COVID-19 cases showed that COVID-19 infection was most prevalent in patients with lymphoma (28.5 %), multiple myeloma (18 %), and AML (13.1 %). The findings here showed a similar distribution to anecdotal data and clinical experience, suggesting that COVID-19 infection is more common in patients with lymphoma and AML. Although the patients in the current study showed a similar distribution in a subtype of HM, the mortality observed in the current study differed from previous reports. According to the American Society of Hematology (mortality among patients with HM that were infected with COVID-19 reached a high of 13.8 %–39 % in June 2021).^10^ In the current study, a total of five patients died, equivalent to a mortality rate of 7.81 %. This mortality rate is lower than the overall mortality rate reported in the literature and was likely due to early intervention with anti-COVID-19 viral drugs in the study.

The mortality benefits of nirmatrelvir-ritonavir and azvudine may result in their direct effects on viral replication. The SARS-CoV-2 virus releases RNA upon entry into cells, and this RNA is subsequently translated into large replicase polyproteins that use nucleoside and nucleotide RNA for replication.^11^ Nirmatrelvir-ritonavir inhibits the major proteases of SARS-CoV-2 (Mpro), also known as 3C protease (3CLpro) or nsp5 protease. 3CLpr is spliced and inserted in the virus's own code. As 3CLpr is the main protease of the RNA, inactivation of 3CLpr inhibits viral replication. In vitro experiments have confirmed that nirmatrelvir-ritonavir associates with 3CLpr after cell entry, and X crystallography studies have found that nirmatrelvir-ritonavir can directly bind to the SARS-CoV-2 3CLpro active site to prevent the replication of viral particles^12^ Azvudine is the first dual-targeted nucleoside drug that targets HIV^13^ and HBV,^14^ with a phase II clinical trial (GQ-FNC-201) showing that azvudine may be effective in treating HIV. More recently, azavudine has shown potent antiviral activity against HCoV-OC43 and SARS-CoV-2 in vitro (unpublished results). Thus, the authors speculated that azvudine may have anti-COVID-19 effects. It has previously been inferred that both nirmatrelvir-ritonavir and azvudine can ultimately achieve the same inhibition of SARS-CoV-2 virus replication despite their different sites of action. However, a single-center cohort study of patients with COVID-19 who received nirmatrelvir-ritonavir or azvudine showed that patients receiving nirmatrelvir-ritonavir had faster viral suppression, earlier negative RT-PCR conversion, and more advantageous viral suppression at the beginning of hospitalization compared to those receiving azvudine.^15^ The results of the present study differ from these previous results, as there were no significant differences between the nirmatrelvir-ritonavir and azvudine groups in terms of first negative nucleic acid test time, hospitalization time, and the rate of conversion of mild or moderate disease to severe disease in the current study. These differences in study results in the previous and the current study may be due to the fact that here patients with HM and COVID-19 were studied, and some patients had received chemotherapy within a month prior to the positive nucleic acid test. Additionally, antiviral drugs may interact with anticancer drugs. Differences in baseline characteristics between treatment groups or small sample size may have also affected the results of the present study, as 40.9 % of the patients with severe COVID-19 infection were enrolled in the nirmatrelvir-ritonavir group, whereas only 23.5 % were enrolled in the azvudine group.

Although there was no difference in time to the first negative nucleic acid test between treatment groups, there was a significant difference in time to first negative nucleic acid test in the nirmatrelvir-ritonavir group (23.5 vs. 34 days, p = 0.015) and in the azvudine group (20 vs. 34 days, p = 0.009) when compared to the observation group. Collectively, these data suggest that nirmatrelvir-ritonavir and azvudine have a similar clinical efficacy in the treatment of COVID-19 in patients with HM, and that both interventions can reduce the time to the first negative COVID-19 nucleic acid test to some extent.

Because HM and recent chemotherapy have been associated with poor outcomes among patients with cancer and COVID-19, the anti-inflammatory effects of nirmatrelvir-ritonavir and azvudine were determined via the expression levels of IL-6, PCT, CRP, and various serological laboratory indicators. CRP (p < 0.001) and IL-6 (p = 0.033) were significantly reduced in patients treated with nirmatrelvir-ritonavir and azvudine. CRP was also significantly reduced in the observation group after symptomatic treatment (p = 0.028), but the degree of CRP reduction was not as great as that observed in the medication treatment groups. No significant differences were observed in serum leukocyte, lymphocyte, and neutrophil levels pre- and post-treatment in patients with HM. This is most likely because patients with HM are usually immunodeficient as a result of the malignancy itself in addition to anticancer treatments or as a result of procedures, such as hematopoietic stem cell transplantation.

Available data suggest that the anti-COVID-19 vaccine is significantly less effective in eliciting an immune response in patients with HM than in the general population,^10^ which may also be partially due to the patients’ immunocompromised states. The present data show that the conversion rate of mild or moderate disease to severe disease was 23.1 % (n = 6) in the unvaccinated population and 18.4 % (n = 7) in the vaccinated population. There was also no statistically significant difference in the conversion rate of mild or moderate disease to severe disease in patients who had or had not received the neo-crown vaccine (HR = 1.714, 95 % CI 0.482–6.099, p = 0.405). In addition, among cancer patients, HM, liver disease, and higher ECOG scores were associated with poorer prognosis,^16^ Higher ECOG scores (HR = 2.284, 95 % CI 1.036–5.038, p = 0.041) and liver disease (HR = 25.281, 95 % CI 2.225–287.193, p = 0.009) were independent predictors of the conversion rate of mild or moderate disease to severe disease in patients with COVID-19 and HM. A previously published large study suggests that recent systemic chemotherapy may not affect mortality and that anticancer therapy should be offered to patients with hematologic diseases requiring urgent treatment despite the risk of COVID-19 exacerbation.^10^

Age may also be strongly associated with the conversion rate of mild or moderate disease to severe disease and mortality.^16^^,^^17^ However, age was not associated with these factors in the present study. These results may be due to the small sample size used in the present study. Further multicenter and large-scale studies are needed to explore the predictive factors affecting the conversion rate of mild or moderate disease to severe disease in patients with HM and COVID-19. In addition, the present study has some limitations, including the following: 1) The three groups of patients were not equally balanced at baseline; 2) The follow-up period was relatively short for patients and may not have been sufficient to collect adverse events that occurred; and 3) Some patients were not on antivirals during the initial phase of their COVID-19 infection.

This study has several noteworthy advantages: it consisted of patients with various types of HM, whereas previous studies of antiviral drugs excluded patients with malignancies, autoimmune diseases, and those who had taken immunosuppressive drugs within three months of admission. The antiviral medication azvudine was approved by the National Health Administration for use in adult patients with COVID-19 in 2022 and is the first domestically produced oral antiviral drug in China. Thus, there have been few clinical trials using azvudine to date. This study also included patients based on inpatient rather than insurance claims data. This allowed us to collect more pre- and post-medication laboratory values. Finally, confounding variables were controlled for by using a multivariate Cox regression model.

### Statement of ethics

All procedures performed in the study were a part of the routine clinical practice and were conducted in accordance with the Declaration of Helsinki and Good Clinical Practice guidelines. The study followed STROBE reporting guidelines and was approved by the Ethics Committee of the Affiliated Hospital of Guilin Medical University. Informed consent was obtained from each study participant (reference number 2023QTLL-19). Patients provided written informed consent.

## Data availability statement

All data generated or analyzed during this study are included in this article. Further inquiries can be directed to the corresponding author.

## Author's contributions

Xiaotao Wang, Wei Chen, Zheng Zeng: Designed the study.

Xiaotao Wang, Wei Chen, Zheng Zeng, Fangyuan Li, MingLi Zhong, Li Jing, Lin Zhu: Performed clinical evaluation and analyzed patients’ data.

Xiaotao Wang, Wei Chen, Zheng Zeng, Fangyuan Li: Analyzed and interpreted data.

Zheng Zeng, Fangyuan Li, MingLi Zhong: Performed statistical analysis.

Xiaotao Wang, Wei Chen, Zheng Zeng, Fangyuan Li: Wrote the manuscript.

Xiaotao Wang, Wei Chen, Zheng Zeng, Fangyuan Li, MingLi Zhong, Li Jing, Lin Zhu: Edited the manuscript.

## Funding

This work was supported by the National Natural Science Foundation of China (grant number 82060044); the Natural Science Foundation of Guangxi Province (grant number NO. 2020GXNSFBA297005, 2020GXNSFAA159018).

## Conflicts of interest

The authors declare no conflicts of interest.

## References

[1] Guan WJ, Ni ZY, Hu Y, Liang WH, Ou CQ, He JX (2020). Clinical characteristics of Coronavirus Disease 2019 in China. N Engl J Med.

[2] Han J, Liu Y, Yang S, Wu X, Li H, Wang Q. (2021). COVID-19 infection in adult patients with hematological malignancies: a european hematology association survey (EPICOVIDEHA). J Hematol Oncol.

[3] Cao Z, Gao W, Bao H, Feng H, Mei S, Chen P (2023). VV116versus Nirmatrelvir–Ritonavir for oral treatment of COVID-19. N Engl J Med.

[4] National Medical Products Administration. China Grants Conditional Approval for Pfizers Oral COVID-19 Drug; 2022.

[5] National Medical Products Administration (2022).

[6] Ma Q, Xie Y, Wang Z, Lei B, Chen R, Liu B (2021). Efficacy and safety of ReDuNing injection as a treatment for COVID-19 and its inhibitory effect against SARS-CoV-2. J Ethnopharmacol.

[7] Hu K, Guan WJ, Bi Y, Zhang W, Li L, Zhang B (2021). Efficacy and safety of Lianhuaqingwen capsules, a repurposed Chinese herb, in patients with coronavirus disease 2019: A multicenter, prospective, randomized controlled trial. Phytomedicine.

[8] National Health Commission (2019).

[9] Lin Y, Wu F, Xie Z (2020). Clinical study of artesunate in the treatment of coronavirus disease 2019. Zhonghua Wei Zhong Bing Ji Jiu Yi Xue.

[10] Infante MS, Salmanton-García J, Fernández-Cruz A, Marchesi F, Jaksic O, Weinbergerová B (2022). B-cell malignancies treated with targeted drugs and SARS-CoV-2 infection: A. Eur Hematol Associat Survey (EPICOVIDEHA). Front Oncol..

[11] Mandal A, Jha AK, Hazra B. (2021). Plant products as inhibitors of coronavirus 3CL protease. Front Pharmacol.

[12] de Vries MD, Mohamed AS, Prescott RA, Valero-Jimenez AM, Desvignes L, O'Connor R (2021). A comparative analysis of SARS-CoV-2 antivirals characterizes 3CL pro inhibitor PF-00835231 as a potential new treatment for COVID-19. J Virol.

[13] Tyack PL, Calambokidis J, Friedlaender A, Goldbogen J, Southall B, Formal comment on Schorr GS, Falcone EA, Moretti DJ (2014). Andrews RD (2014) first long-term behavioral records cuvier's beaked whales (Ziphius cavirostris) Reveal record-breaking dives. PLoS One.

[14] Zhou Y, Zhang Y, Yang X, Zhao J, Zheng L, Sun C (2012). Novel nucleoside analogue azavudine is effective against both wild-type and lamivudine-resistant HBV clinical isolates. Antivir Ther.

[15] Gao Y, Luo Z, Ren S, Duan Z, Han Y, Liu H (2023). Antiviral effect of azvudine and nirmatrelvir-ritonavir among hospitalized patients with COVID-19. J Infect.

[16] Grivas P, Khaki AR, Wise-Draper TM, French B, Hennessy C, Hsu CY (2021). Association of clinical factors and recent anticancer therapy with COVID-19 severity among patients with cancer: a report from the COVID-19 and Cancer Consortium. Ann Oncol.

[17] Vijenthira A, Gong IY, Fox TA, Booth S, Cook G, Fattizzo B (2020). Outcomes of patients with hematologic malignancies and COVID-19: a systematic review and meta-analysis of 3377 patients. Blood.

